# The taphonomic impact of scavenger guilds in peri-urban and rural regions of central and southern Alberta. Part II - Dispersal patterns of forensically relevant vertebrate scavengers

**DOI:** 10.1016/j.fsisyn.2025.100649

**Published:** 2025-11-21

**Authors:** Yvonne Kjorlien, Dennis Dalziel, Christopher J. Watson, Shari L. Forbes

**Affiliations:** a Office of Research, Scholarship and Community Engagement, Mount Royal University, 4825 Mount Royal Gate, Calgary, T3E 6K6, Alberta, Canada; b Edmonton Police Service Canine Unit, 9620 - 103A Avenue, Edmonton, Alberta, T5H 0H7, Canada; c Parks Canada, Jasper Field Unit, PO Box 10, Jasper, T0E 1E0, Alberta, Canada; d Department of Chemistry and Biochemistry, University of Windsor, 401 Sunset Ave. Rm. 220 Essex Hall, Windsor, Ontario, N9B 3P4, Canada

**Keywords:** Forensic taphonomy, Vertebrate scavenging, Decomposition, Skeletal elements, Dispersed remains

## Abstract

Recovering forensic evidence in dispersed scenarios is challenging because of the taphonomic influence of scavengers. This study aimed to observe how remains were dispersed by the scavenger guilds identified in Part 1 (Forbes et al., 2024) of a Canadian study, which include bears, canids, and magpies, and other possible environmental factors. Clothed pig carcasses (*Sus scrofa*) were placed in open- and closed-canopy habitats of peri-urban and rural regions of two major cities in Alberta (Calgary and Edmonton). Four carcasses were deposited in each city in August 2021 and again in 2022, and a search to document and recover the remains was conducted 10–12 months after deposition. The mean recovery rates of skeletal elements were 22.5 % (2.0 %–66.9 %). Calgary rural open and closed environments had the largest maximum dispersal distances (60–70 m) which may be associated with the presence of grizzly bears and canid scavengers (coyotes and semi-wild dogs). There was no statistical difference in dispersal distances between open and closed habitats. Limbs were the most common skeletal element dispersed furthest. The results offer new and relevant information to organizations tasked with searching for human remains, especially remains that may have been scavenged and scattered from the original deposition site in an Albertan landscape.

## Highlights

• Large mammalian scavengers can accelerate hard tissue loss in Alberta, Canada.
• Continued skeletal remains dispersal over time with 22.5 % average recovery rate.
• Canids account for large dispersal distances, even when other mammals scavenge.
• Limbs were the most common skeletal element dispersed furthest.
• Rural area scavenger diversity influences recovery rates and dispersal distances.

## 1 Introduction

One of the greatest challenges in the search and recovery of human remains is predicting the dispersal of the remains so that recovery rates may be maximized. Knowledge of dispersal patterns can inform search methods, thereby increasing the likelihood that potential forensic evidence will be recovered. Knowledge of the taphonomic agents acting on the remains and their impact on dispersal can inform a search and increase the potential to locate and return more of the deceased's remains to the next of kin. Scavengers are often the greatest contributor to the disarticulation, dispersal, and removal of remains and personal effects [[1], [2], [3]]. Research on scavenger behaviour is becoming more common (i.e. [[4], [5], [6]]); however, research on the subsequent dispersal of skeletal material is still lacking.

In North America, the dispersal of remains due to taphonomic processes has been observed or studied in numerous locations, including Washington [7,8], Arizona [9,10], Virginia [11], and Florida [12,13]. Research has broadened to include international locations, such as South Africa [5,[14], [15], [16], [17]], East Africa [18], South America [[19], [20], [21]], Europe [[22], [23], [24], [25]] and the UK [4] to recognize that taphonomic agents may differ over time and space. However, even with this broadening of the knowledge base, there remain limitations in the literature which preclude elucidating patterns in the dispersal of remains. Forensic cases report cross-sectional or anecdotal dispersal information, often with the dispersal of the remains as a peripheral focus (i.e. [2,[25], [26], [27], [28]]). Few taphonomic experimental studies focusing on dispersal patterns for forensic contexts have been conducted using a sample size larger than two ([i.e., 9, 11–12]) or for longer than a few months (i.e. [1,4]). One experimental study [29] has been published for Canada that uses a larger sample size (n = 24) and focuses on the dispersal of remains over two summer periods of just over three months each. To date, limited literature has been published that investigates possible correlations between dispersal patterns due to scavenger behaviour and taphonomic influences, such as topographic features (i.e., slopes, roads, fence lines, human activity) [13] and local environmental attributes (i.e., climate, humidity, vegetation). The current study was conducted to add to the knowledge base of taphonomic factors that may impact dispersal patterns in populated regions of Canada.

This study was designed in conjunction with the Calgary and Edmonton Police Canine Units to provide new information to human remains detection (HRD) dog handlers that could assist with their searches for human remains. Overall, the two aims of the study were to identify the scavenger guilds in specific areas, and how they impact the loss of soft and hard tissues. Part 1 of this study [30] identified the scavenger guilds of bears, canids, and magpies as the most prevalent animals scavenging upon the carcasses. We determined the scavenger guilds’ variability based on urban proximity and habitat. Part II, reported herein, outlines observations of the dispersal of remains with respect to those scavenger guilds and other possible environmental factors.

## 2 Methods

### 2.1 Experimental locations and human analogues

The study was conducted across a two-year period (2021–22 and 2022–23) to investigate inter-year variability. Personal communication from local law enforcement agencies demonstrated the limited comparative use of short-term experimental trials (i.e., 1–3 months, or one season) to investigative files of individuals who were likely to have been in an outdoor context for extended periods. Hence, all trials were left *in situ* for approximately 10–12 months, from August to the following June–August.

The study comprised 16 trials in which 16 pig carcasses were placed near two major cities of Alberta, Canada (Calgary and Edmonton) based on the frequency with which the police partners are asked to search for scavenged human remains. Each city included two proximity locations, a peri-urban location and a rural location, to determine any patterns of urban proximity with scavenging activity. Each proximity location included two habitats: an open habitat (no tree canopy) and a closed habitat (a closed tree canopy), as in previous Canadian scavenging studies [[30], [31], [32]]. In each year, four pig carcasses were placed in one of four proximity and habitat combinations: 1) open, peri-urban, 2) open, rural, 3) closed, peri-urban, and 4) closed, rural. Each city had a pig carcass placed in each of these four proximity-habitat combinations, and this was repeated in the second year, for a total of 16 carcasses or trials. Carcasses were placed in the summer (August) with the search and recovery of remains conducted the following summer (June–August), 10–12 months after initial deposition. The dispersal of skeletal elements from each carcass was quantified during the search. The locations within Calgary and Edmonton were changed between 2021–22 and 2022–23 to investigate comparable peri-urban and rural regions in proximity to each city, but also to avoid learned behaviour from local scavengers of the previous year (see Ref. [30]). This paper uses “carcass” to denote a whole body whereas parts of the carcass are referred to as “remains” or the specific part of the carcass, such as skeletal element or soft tissue.

The vegetation in the study area was broadly similar across the sites (see Ref. [30] for more details). Within each site, the distance between open and closed habitats ranged from approximately 200 m to 2 km depending on land availability. The sites were chosen as they represented typical terrain in these areas where the HRD dog handlers had previously searched for human remains.

In Canada, the use of human analogues is deemed necessary to investigate the local scavenger guilds in regions such as the province of Alberta where human remains cannot be studied. This study used domestic pig carcasses (*Sus scrofa domesticus*, Suidae) [33]. For each trial, a pig carcass weighing ∼200–250 lbs (approx. 90–113 kg) was used. Pig carcasses were purchased from local farmers as excess food stock (which could not be sold for human consumption) and thus did not require animal ethics approval through the lead organisation. All subjects had been killed the morning of collection using a penetrating captive bolt [34]. All carcasses were transported in individual body bags. Carcasses for each city were deposited on the same day they were killed. All carcasses were clothed in human clothing, either a dress or shirt and shorts to approximate real situations during summer months.

Each carcass was anchored to a nearby tree using wire cable wrapped around the upper limbs and torso as this has previously been shown to have minimal impact on the scattering of remains (see Ref. [25] for more details). Keyes et al. [16,17] also anchored carcasses and found no impact on the scavenging and scattering of the remains.

Lotek Ultimate VHF Collars (typically used for terrestrial wildlife telemetry tracking and monitoring [35]) were added to the 2022 trials to help provide further dispersal information. Each pig was fitted with 1–2 collars (weight approx. 5 g). A drill was used to create a hole through soft and hard tissue on a limb or snout in the carcass to ensure it would remain attached to the skeletal element after decomposition, disarticulation, and gnawing. The collar was threaded through the hole and secured with a zip tie. The tag frequency and its location on each carcass was recorded.

### 2.2 Observations and recordings

For the 2021–22 trials, two trail cameras were installed on a nearby tree to monitor continuously the scavenging activity on and around each pig carcass. For the 2022–23 trials, a third camera was added to each site to capture video recordings in addition to the still images of the other two cameras (see Ref. [30] for details on each camera).

**Fig. 1:**
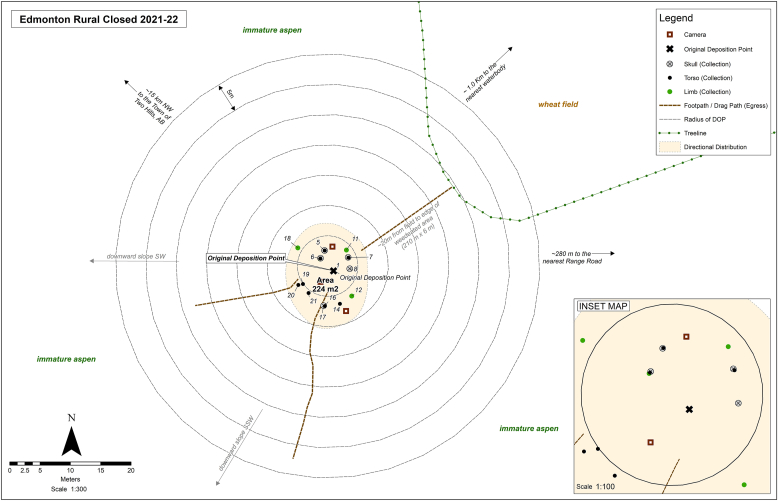
An example of a digitized sketch map produced of an Edmonton rural trial.

Each carcass was labelled according to the adjacent city, the habitat, and the proximity of the city, such as Edmonton, rural open (as in “open” canopy). A sketch map was created for each carcass at the time of deposition. The use of a Total Station for mapping was deemed unsuitable at the time of the study because of the presence of tree canopy in half the locations (also shown by Ref. [36]). A handheld Garmin GPS was used to record the Universal Transverse Mercator (UTM) for coordinates of each carcass, using the North American Datum of 1984 (NAD 84). The tree canopy also affected the accuracy of the handheld GPS unit, so a cloth tape measure and compass were also used to document and map the dispersed remains. The sketch map [34] recorded the label and position of the carcass (the original deposition position, ODP), approximate position of the cameras in relation to the carcass, the UTM coordinates of the carcass, magnetic north, the clothing of the carcass, and, if a tracker was used, the frequency of the tracker and its position on the carcass. Each map also documented the habitat of each carcass, including approximate distance to treelines, known water bodies, roads, fences, type of vegetation, pre-existing game trails, and the egress path used to deposit the carcass (see Fig. 1 example).

### 2.3 Search and recovery

Each search was conducted by the same two forensic taphonomists and two canine handlers from the respective police partner locations who had comparable search knowledge and expertise. Many recent studies have shown no consensus on which search method to use for dispersed remains [[37], [38], [39], [40]]. The link method, however, has been shown to be more efficient for scatter patterns [4,41,42]. The link method, similar to winthropping [43], allows the searcher to adjust their search direction based on cues in the environment, such as tracks or game trails [4]. This method was used to cover a maximum search area of approximately 100 m radius from the ODP, unless using the VHF antenna to find the radio tags. When the antenna was used to find radio tags, the maximum search area was up to 400 m from the ODP. At the completion of the study, a forensic anthropologist re-visited the sites to confirm no additional skeletal elements were missed within the 100 m search areas.

At the search and recovery for each site, the sketch map was revised to include the position of any dispersed remains and new game trails (Fig. 1). All remains, including clothing, were indicated with survey flags and the flags were numbered [29,44]. Initially, the distance and direction of dispersal from the ODP was recorded using a GPS. Due to the error associated with low-precision GPS, the distance of all remains (bone and soft tissue) and clothing were additionally measured with a cloth measuring tape and compass from the ODP to ensure accuracy. The remains were then recorded on the map and listed according to the flag number. A VHF antenna was programmed with each radio tag frequency for each carcass. Radio tags for the eight 2022–23 carcass were located, if possible, and the UTM coordinates of the tags were documented on the map. All remains discovered during each search were collected, identified, and inventoried according to each site.

Recovery rates are based on the percentage of the total number of skeletal elements recovered. For the purposes of this study, the total number of skeletal elements in a single pig carcass was set at 181 (Table 1). Previous publications [[45], [46], [47]] have varying total skeletal counts for domestic pigs because domestication can influence the number of certain skeletal elements, such as the number of ribs. The total for this study was based on the following:

● many of the pigs used in this study were subadults even though their precise age was unknown and many of the carcasses included unfused epiphyses. These unfused epiphyses were not included in the final skeletal remains counts, unless they were morphologically distinct and could represent the entire skeletal element (e.g., an unfused femoral head could represent a femur if no other portion of the femur is recovered).
● teeth were excluded in the final skeletal remains counts.
● the skull was considered to comprise five components: the cranium, right and left mandible, and right and left maxillae. If a portion of any of these five components were discovered or recovered, it counted as representing that component. The cranium was only counted as one element as previous taphonomic studies have only referred to “head” or “skull” (e.g. Refs. [18,19]) and reducing the cranium to its constituent bones would not add value to the scope of this study.
● ribs counts were based on 14 on each side, for a total of 28.
● vertebrae counts were based on 7 cervical, 14 thoracic, 6 lumbar, 4 sacral, and 18 coccygeal.
● one sternum
● limbs were counted as: 2 ulna, 2 radii, 2 humerus, 2 femur, 2 tibia, 2 fibula, 2 scapula, 6 os coxae, 14 tarsals, 12 carpals, 16 metacarpals/tarsals, and 26 phalanges.

**Table 1:** Number of skeletal elements in *Sus scrofa* [27,28].

Skeletal Element	This Study
Skull	5 = mandible (2), maxilla (2), cranium (1)
Vertebrae	49 = cervical (7), thoracic (14), lumbar (6), sacral (4), coccygeal (18)
Ribs	28 = 14 on each side
Sternum	1
Limbs	98 = ulna (2), radius (2), humerus (2), femur (2), tibia (2), fibula (2), scapula (2), os coxae (6), tarsals (14), carpals (12), metacarpals/tarsals (16), phalanges (36)
**Total**	**181**

One carcass's remains in 2022 was comparatively undispersed at the end of the study and was used to form an estimate of the number of skeletal elements of the pig carcasses used in this study. The number of skeletal elements is outlined in Table 1.

### 2.4 Data collection and analysis

Decomposition stages were not estimated for this study due to rapid desiccation of the remains at all sites and the difficulty of scoring decomposition when regions of the carcass are being consumed by vertebrate and invertebrate scavengers [30]. The minimum number of elements (MNE) for each carcass involved counting each identifiable skeletal element and removing any redundancies (e.g., one left humerus shaft and one left humerus distal epiphysis recovered at one carcass trial would be counted as one left humerus) [19]. Skeletal elements were broken into three groups: 1) skull, including the cranium, mandible, and maxilla; 2) vertebrae, ribs, and sternum; and, 3) limbs, including scapula and os coxae. The scapula and os coxae were included with the limbs because they have been shown to disarticulate from the skeleton sooner than vertebrae and ribs, and may be removed from a carcass by a scavenger as a unit with the associated limb [2,48]. Each identifiable skeletal element was then assigned to one of the three skeletal groups.

In the analysis, an arbitrary delineation was set at a radius of 10 m from the ODP to highlight which skeletal elements remained nearest the ODP. Percentage of the total skeletal count (n = 181) for each skeletal element group was also calculated. The data were imported into R software [49] for processing and analysis of correlations between dispersal patterns and environmental factors, including the presence and interaction by recorded scavengers. Dispersal metrics at each site are reported by MNE, as well as i) mean dispersal (the mean distance that skeletal elements were located), ii) maximum dispersal (the furthest distance that skeletal elements were discovered), and iii) recovery rate (the percentage of each carcass skeleton that was recovered). Due to the non-normality of data, comparative non-parametric statistical analysis of dispersal distance within different groups was conducted (e.g. Wilcoxon rank-sum, Kruskal-Wallis), with the test statistic and p-value reported.

Discussions regarding the identification of scavenging marks, survivability and recoverability of skeletal elements [48,50,51], and the differences of skeletal elements between *Sus scrofa* and *Homo sapiens* are beyond the scope of this study.

## 3 Results

### 3.1 Scavenger guilds

A diversity of species were identified across all trials [30], however the Calgary peri-urban, Edmonton peri-urban, and Edmonton rural scavenger guilds were dominated by coyote (*Canis lantrans,* Canidae) and the black-billed magpie (*Pica hudsonia,* Aves). While the black-billed magpies often fed in groups and for extended periods of time during the day, their impact in terms of soft tissue removal was minimal and the majority of soft tissue loss and hard tissue dispersal was the result of coyote scavenging. In contrast, the predominant scavengers at the Calgary rural sites were grizzly (*Ursus arctos horribilis,* Ursidae) and black (*Ursus americanus,* Ursidae) bears. Coyote, semi-wild dogs and black-billed magpies also scavenged, however their feeding was considerably reduced compared to the other sites, likely due to presence of bears in the area. Grizzly bears were only observed at the Calgary rural sites since their range does not extend to the peri-urban regions of Calgary or any region of Edmonton.

### 3.2 Recovery rates

On average, 40.7 skeletal elements were recovered per carcass, which equates to 22.5 % of a carcass’ skeleton. The percentage of skeletal elements recovered varied between 2.0 % (2021 Edmonton peri-urban closed) and 66.9 % (2022 Calgary peri-urban closed). For 15 of the 16 trials, less than half of the carcass skeleton was recovered. In only one trial (2022 Calgary peri-urban closed) was more than half recovered (66.9 %). This was also the trial that was used to establish a baseline skeletal element count for this study.

### 3.3 Dispersal of skeletal elements by region

**Fig. 2:**
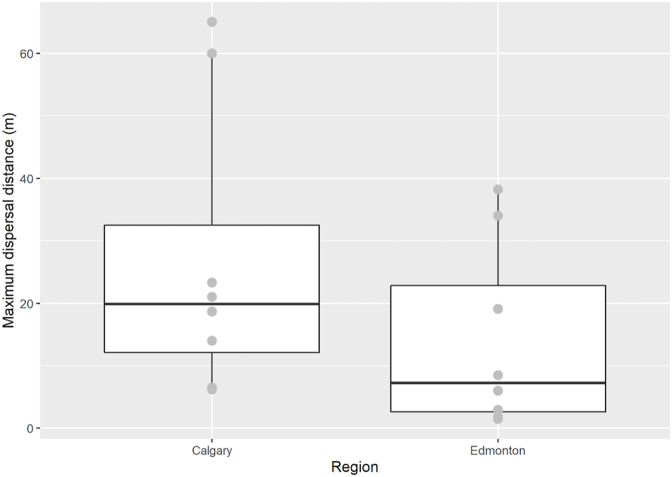
An overview of the maximum dispersal distance of skeletal elements from ODP in the Calgary and Edmonton trials. Boxplots represent first to third data quartiles with the mean of individual sites represented by grey points.

The Calgary trials demonstrated a higher average maximum dispersal (26.8 m; Fig. 2) and a higher mean dispersal (8.8 m) than the Edmonton trials (16.3 m and 5.0 m, respectively). There was high variability within these data: two of the Calgary trials (2021 Calgary rural open and closed) had large dispersal radii (65.0 m, 60.0 m, respectively), whereas the other Calgary trials had a maximum dispersal distance of no more than 21.0 m. All Edmonton trials had a maximum dispersal distance of 38.0 m or less, with three trials dispersing less than 10.0 m.

### 3.4 Dispersal of skeletal elements by habitat type and urban proximity

**Fig. 3:**
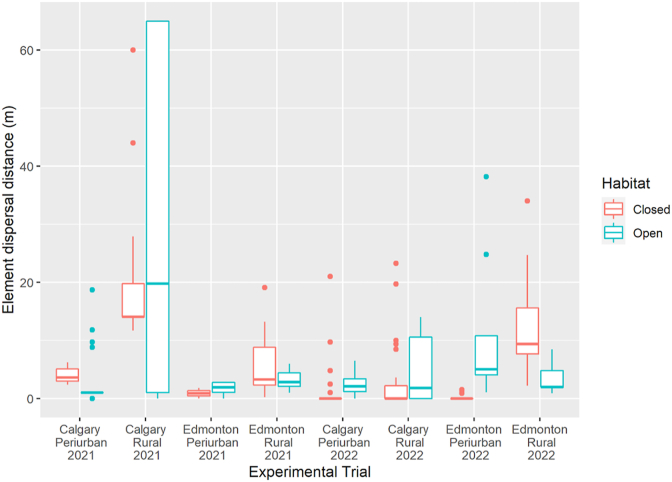
Dispersal distances of elements recovered from the 2021 and 2022 trials in Calgary and Edmonton. Boxplots represent the first to third data quartiles. Points indicate statistical outliers.

Dispersal distances were not impacted by habitat, with similar metrics observed for both open (mean dispersal = 7.07 m; average maximum dispersal = 22.0 m) and closed (mean dispersal = 6.78; average maximum dispersal = 21.5 m) sites. Open sites had a greater variance than closed sites (see Fig. 3), however, neither the mean nor the maximum dispersal were significantly different between open and closed sites (Wilcoxon rank-sum test, mean dispersal, W = 40, p = 0.4418; maximum dispersal, W = 31, p = 0.9591).

Dispersal distances were greater at rural sites than peri-urban sites. Mean dispersal distance at rural sites was 11.5 m compared to 2.3 m at peri-urban sites, which was significantly different (Wilcoxon rank-sum test, mean dispersal: W = 5, p = 0.0029). The average maximum dispersal distance was 30.8 m at rural sites compared to 12.3 m at peri-urban sites, though non-significant at p = 0.05 (W = 15, p = 0.0083).

There was no dispersal pattern demonstrating deliberate movement away from the nearest roadway or towards or away from the nearest waterbody. Possible coyote den sites were located at two trials (2021 Calgary rural closed and 2022 Edmonton rural closed), but it was not possible to determine if the dens were in active use by the coyotes scavenging the nearby carcass. Remains were frequently recovered from existing and newly created game trails.

### 3.5 Dispersal distance of skeletal elements by bone type

**Fig. 4:**
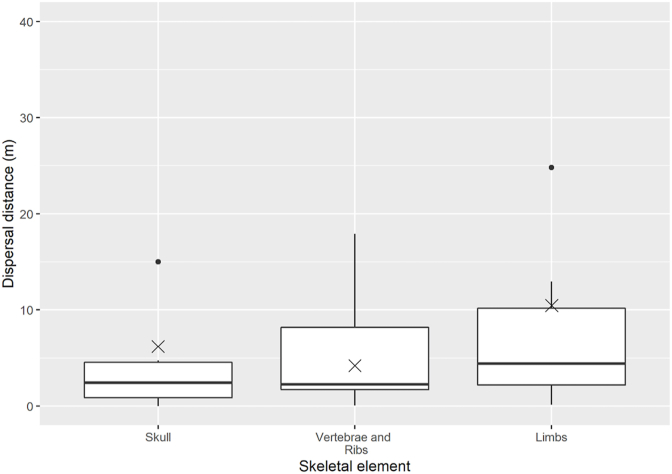
An overview of average maximum dispersal distances across all 16 sites of the skeletal groups: limbs (including os coxae and scapula), skull, vertebrae and ribs for the 2021 and 2022 trials combined in Calgary and Edmonton. Boxplots represent the first to third data quartiles, mean value is represented by (X).

Fig. 4 shows the overall dispersal distances of the three skeletal elements groups: 1) skull, 2) limbs with scapula and os coxae, and 3) vertebrae and ribs. Across all the carcasses, the limb group elements, on average, tended to be dispersed further (mean = 10.5 m) than those of the skull group (6.2 m) or the vertebrae and ribs group (4.2 m), though differences between groups were not found to be significant (Kruskal-Wallis test, χ2 = 42, p = 0.4274). The vertebrae and ribs group represented the largest portion of skeletal elements recovered from within a 10-m radius of the ODP. Sometimes vertebrae and/or ribs would be recovered from within the area of body staining and dead vegetation that marked the ODP. When portions of the skull were recovered, they were mostly found within 10 m of the ODP.

### 3.6 VHF radio tags

Lotek Ultimate VHF Collars were inserted into bones on all eight carcasses in the 2022–2023 trials to provide further information on maximum dispersal. Radio tags associated with only four of the carcasses were located (Table 2).

**Table 2:** Lotek Ultimate VHF Collars recovered during the 2022 trials in Calgary and Edmonton.

Trial	Skeletal Element of Tracking Device	Distance Recovered (m)
Edmonton Peri-urban Open	femur	201
Edmonton Rural Closed	unknown limb	140
Calgary Peri-urban Closed	ulna	@ ODP
Calgary Rural Closed	femur	@ ODP

**Fig. 5:**
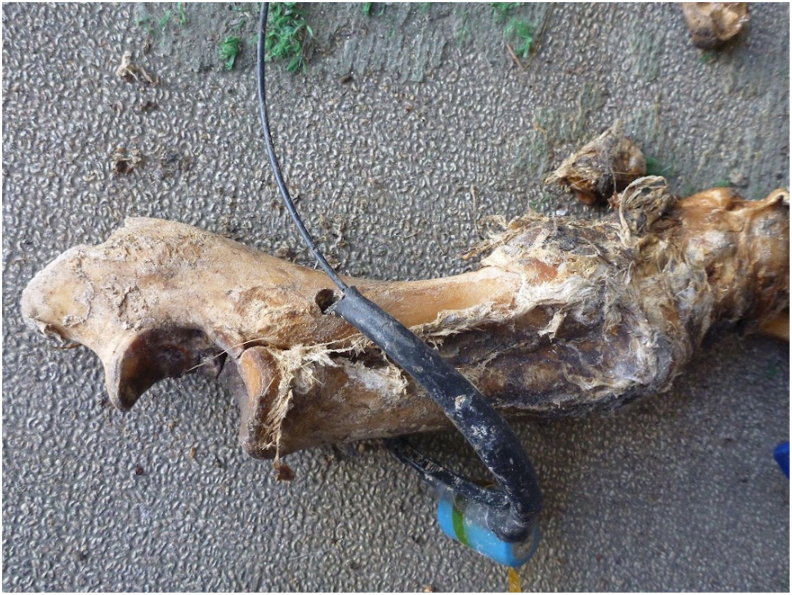
Ulna of one of the Calgary 2022 peri-urban trials in which the tracking device was looped through and recovered intact from the ODP.

At the Edmonton rural closed trial, the signal was followed into a low-lying area 140 m east of the ODP. The antenna indicated the presence of the radio tag; however, the tag and its associated skeletal element could not be found, likely due to burial under dense vegetative matter. At the Edmonton peri-urban open trial, the radio tag and its associated femur was recovered 201 m along the embankment of the North Saskatchewan River. The collar was still looped through the hole in the femur shaft. At the Calgary peri-urban trials, both radio tags were recovered attached to their associated skeletal elements within 2 m of the ODP of each respective trial (Fig. 5).

Dispersal data of these skeletal elements containing radio tags were not included in the data analysis due to their limited recovery, and because they did not influence the overall dispersal patterns observed across all trials. Only two tags were recovered beyond the maximum 70 m dispersal radius of the other remains. However, it is important to note that the Lotek Ultimate VHF Collars were valuable in highlighting greater recovery distances up to 201 m from the ODP.

## 4 Discussion

The aim of Part II of the study was to demonstrate how scavengers impact the loss of hard tissues (skeletal elements) through possible dispersal patterns. All trials were commenced during the summer months, allowing for inter-year comparison.

### 4.1 Dispersal of skeletal elements by region

Mean dispersal and maximum dispersal distances were higher in Calgary trials compared to Edmonton trials, predominantly influenced by the presence of grizzly bears at the Calgary rural sites with subsequent canid scavenging [30]. It is suspected that higher dispersal distances at these sites was the result of initial disarticulation and soft tissue consumption by grizzly bears, which facilitated dispersal activity by coyotes and semi-wild dogs after the grizzly bears had finished scavenging. However, this activity cannot be confirmed as the carcasses were already dragged out of frame by grizzly activity by that time. All Edmonton trials were dominated by coyote scavengers that would feed either alone, in pairs, or in groups of up to five members [30].

### 4.2 Dispersal of skeletal elements by habitat and urban proximity

Part 1 of this study [30] revealed that no scavenging species showed a preference for open or closed trials. This was further confirmed in Part II of the study whereby no significant difference was observed for the average maximum dispersal distance, i.e., 21.5 m for closed sites and 20.0 m for open sites. Despite this similarity, open sites did have a greater range of dispersal suggesting in some circumstances that greater dispersal is possible. It was noted through video imagery that coyote would generally attempt to move the carcass from the open area to a more densely vegetated (closed) area where possible. This aligns with the findings of Grubb & Ellis [52], who noted that coyotes would often drag the carcasses from the original deposition site.

Part I of this study [30] showed that urban proximity in the Calgary region influenced scavenger diversity between the peri-urban and rural sites. Evidence of this scavenger diversity was observed in Part II through the differences in peri-urban and rural dispersal distances and recovery rates (26.8 % and 17.8 %, respectively). Rural sites in Calgary were often visited by bears, which tended to consume most of the soft tissue. This made dispersal of more portable skeletal elements by canids possible.

### 4.3 Dispersal of skeletal elements by bone type

The natural disarticulation, or decomposition of the soft tissue which holds a joint in articulation, may allow a skeletal element or a group of skeletal elements to be transportable by animals or other environmental taphonomic processes [2]. The disarticulation sequence may, therefore, influence what skeletal elements are available to be removed from the ODP and dispersed.

Haglund et al. [2] outlined a consistent disarticulation sequence for *Homo sapiens* when canid scavenging is involved. This disarticulation sequence was based on observations on *Homo sapiens* through Haglund's casework in the US Pacific Northwest and on previous work by Toots [53], Hill [18], and Haynes [54]. A previous study in the Edmonton area [55], where canids are prevalent, showed the similarities between stages developed by Haglund et al. [2] and a possible disarticulation sequence for *Sus scrofa*. These studies (as well as [20,56,57]) show that the vertebral column is the last to disarticulate. As such, vertebrae were much more likely to remain close to the ODP in forensic contexts (and also shown by Refs. [9,16,[58], [59], [60]]). Haglund et al. [2] also showed that the skull was the least likely to indicate the ODP as it was the most likely to be dispersed. Other studies have since shown that the skull's presence near the ODP is not consistent across geographic locations and scavenger guilds (e.g., Refs. [23,25,50,61]. Previous studies (e.g. Refs. [2,18,19,[56], [57], [58], [59], [60]]) demonstrate that appendicular skeletal elements and groups of skeletal elements would disarticulate and disperse first. In the current study, limbs tended to be dispersed further than skull bones or vertebrae and ribs. Previous studies also demonstrate that, because vertebrae and ribs naturally disarticulate later in the sequence, they are the most likely to remain at or near the ODP. In the current study, vertebrae and ribs represented the largest portion of skeletal elements recovered within 10 m of the ODP. Despite the differences in pig skulls and human skulls, the results of the current study suggest that the skull is not a good indicator of the ODP.

Bright [62] and Carson et al. [61] note the important distinction that canids may scavenge, consume, and disarticulate a carcass differently than bears. In the current study, however, canids and grizzly and black bear scavenging influences could be overlapping. Observations of scavenging were limited to those captured by the cameras within the first 2–3 months of the study (Part 1 [30]). It was not possible in this study to distinguish between canid and bear scavenging influences on the maximum or mean dispersal distances or on the disarticulation sequence. It is suspected that scavenger diversity had a cumulative influence on remains disarticulation and dispersal.

Characteristics of the ODP, such as body staining, dead vegetation, and higher frequency of ribs and vertebrae, were observed at nearly all sites. The presence of body staining and dead vegetation indicates that scavenging occurred after the carcass had reached a stage of decomposition that allowed decomposition fluids to stain the ground, kill the vegetation, and for the break-down of tendons, ligaments, and muscles to occur. In the absence of high-intensity early scavenging (i.e., where grizzly bears occurred), disarticulation occurs more readily at later stages of decomposition, leaving behind ribs and vertebrae, while other remains, such as limb elements, could be dispersed. At those sites where scavenging occurred soon after deposition (i.e., 2021 Calgary rural open and closed), less body staining and fewer skeletal elements were discovered.

### 4.4 Dispersal of skeletal elements by scavenger

As discussed in Part 1 [30], the predominant scavengers across all sites except the Calgary rural sites were the coyote (*Canis lantrans*, Canidae) and the black-billed magpie (*Pica hudsonia*, Aves). The predominant scavengers at the Calgary rural sites were grizzly (*Ursus arctos horribilis*, Ursidae) and black bear (*Ursus americanus*, Ursidae). Use of the radio tags showed that remains could be transported by animals up to 200 m from the ODP. Previous studies which include canid scavenging [2,8,23,25] have shown that remains can be dispersed at least 200 m/656 ft or even as far as 185 miles/297.7 km [8].

As the ability to disperse skeletal elements depends upon the size and strength of the animal, the dispersal radius may vary in comparison to the scavenger guilds present. For example, Young et al. [4] found that in the UK the primary scavengers responsible for dispersing remains in their study were the Red Fox (*Vulpes vulpes*) and Eurasian Badger (*Meles melse*). Remains dispersed by fox scavenging activity tended to be within a 45 m radius of the ODP, with some exceptions traveling approximately 100 m. In South Africa, the primary wild scavengers are vultures, with mongoose, genet, civet, warthog and honey badger contributing [16]. Keyes et al. [16] found that the dispersal radius produced mainly by vultures was less than 10 m from the ODP.

Coyotes were observed scavenging at every site, and bears were observed scavenging only at the Calgary sites [30]. The Calgary sites had a higher maximum dispersal radius and also greater diversity in the maximum dispersal radius. The differences in the maximum dispersal radii of the Calgary sites may be a consequence of the cumulative scavenging by both bear and canids.

### 4.5 Limitations of the study and future work

Site mapping and documenting the identity and location of each item found by the search team took considerable time by the forensic anthropologist. Efforts are already being made in our ongoing studies to use technology to map each trial area and the dispersed remains. Researchers and practitioners are already using technology to document sites involving dispersed remains [35,[63], [64], [65], [66], [67]] much the same way that archaeologists are using it to preserve a context that will be destroyed with a search. In future studies, it is hoped that this technology will allow us to capture valuable information that can later be analysed for currently unknown or unobserved patterns.

The VHF radio tags were only incorporated into the 2022–23 trials. Those tags recovered provided anecdotal evidence of a broader pattern of greater dispersal occurring. Of the four devices located, two were located at distances greater than the dispersal pattern observed in non-tagged skeletal elements and lend support for case studies in which skeletal elements were recovered miles or kilometres from the ODP [2,8,23,25]. Future studies using telemetry may substantiate the anecdotal and case study evidence for dispersal distances greater than 200 m from the ODP and indicate additional areas of study.

## 5 Conclusions

Our study has identified overall dispersal patterns for Calgary and Edmonton during 2021-22 and 2022–23 trials. Overall recovery rates of skeletal elements were a mean of 22.5 % with a range of 2.0 %–66.9 %. Negligible difference was observed between open and closed sites, which follows findings in Part 1 that scavenging behaviour was mostly unaffected by habitat, with the possible exception of coyotes moving elements to cover from open sites. Calgary rural sites were found to have the largest maximum dispersal distances, likely because grizzly bear scavengers at these locations made the skeletal elements available to canids for subsequent dispersal. Hence, the difference between Calgary and Edmonton data can be attributed to the presence of larger scavengers, rather than other regional differences (e.g., climate, etc.). The limb group of skeletal elements was the most common group of skeletal elements to disperse the furthest, whereas skull and vertebrae/ribs tended to remain closer to the ODP. Our findings suggest that using telemetry would provide valuable information regarding the likelihood of dispersal beyond 100 m from the ODP. The results offer new and relevant information to organizations tasked with searching for human remains, especially remains that may have been scavenged and scattered from the original deposition site in an Albertan landscape.

## CRediT authorship contribution statement

**Yvonne Kjorlien:** Writing – review & editing, Writing – original draft, Validation, Project administration, Methodology, Investigation, Formal analysis, Data curation, Conceptualization. **Dennis Dalziel:** Project administration, Methodology, Investigation, Conceptualization. **Christopher J. Watson:** Writing – review & editing, Writing – original draft, Validation, Methodology, Formal analysis, Data curation. **Shari L. Forbes:** Writing – review & editing, Writing – original draft, Validation, Project administration, Methodology, Investigation, Funding acquisition, Formal analysis, Data curation, Conceptualization.

## Funding information

The Canada 150 Research Chair in Forensic Thanatology [C150-2017-12] and the Natural Sciences and Engineering Research Council of Canada [RGPIN/6098/2019] supported this research.

## Declaration of competing interest

No potential conflict of interest is reported by the authors.
